# How children make sense of climate change: A descriptive qualitative study of eco-anxiety in parent-child dyads

**DOI:** 10.1371/journal.pone.0284774

**Published:** 2023-04-20

**Authors:** Terra Léger-Goodes, Catherine Malboeuf-Hurtubise, Karen Hurtubise, Kyra Simons, Amélie Boucher, Pier-Olivier Paradis, Catherine M. Herba, Chantal Camden, Mélissa Généreux

**Affiliations:** 1 Faculty of Medicine and Health Sciences, Université de Sherbrooke, Sherbrooke, QC, Canada; 2 Department of Psychology, Université du Québec à Montréal (UQAM), Montréal, QC, Canada; 3 Department of Psychology, Bishop’s University, Sherbrooke, QC, Canada; 4 Centre de Recherche du Centre Hospitalier Universitaire de Sherbrooke (CRCHUS), Sherbrooke, QC, Canada; 5 Department of Psychology, Université de Sherbrooke, Sherbrooke, QC, Canada; 6 Research Center of CHU Sainte-Justine, Montreal, QC, Canada; 7 Institut Universitaire de Première Ligne en Santé et Services Sociaux (IUPLSSS) du Centre Intégré Universitaire de Santé et Services Sociaux (CIUSSS) de l’Estrie, Sherbrooke, QC, Canada; Asia University, TAIWAN

## Abstract

The climate crisis not only has significant impacts on biodiversity and the physical health of humans, but its ramifications are also affecting people’s mental health. Eco-anxiety, or the emotions that emerge with the awareness of climate change and the apprehension of its detrimental effects, has been investigated in adults and adolescents, but much less attention has been given to the impacts on children’s mental health and well-being. Initial evidence confirms that youth are significantly concerned about climate change, but few studies have investigated the resulting emotional responses of children and the role of their parents in tempering these, especially using qualitative methodologies. The present study used a descriptive qualitative design with a convenience sample of parents and child dyads, assessed separately. Children’s (*n* = 15, ages 8–12 years) experiences were explored using semi-structured interviews and their parents’ (*n* = 12) perceptions were captured using a survey with closed and open-ended questions. A reflexive thematic analysis was used to analyze the interview data, and content analysis was used to investigate parent-child experiences. Three themes emerged from the thematic analysis: 1. children’s understanding of climate change, 2. their emotional reaction to climate change, and 3. their coping mechanisms to deal with these emotions. The comparative content analysis revealed that parents who were aware that their children had concerns about climate change, had children who used more adaptive coping mechanisms. The results of this qualitative study contribute to a better understanding of children’s emotional experience of the awareness of climate change in Canada and how they cope with these emotions. Furthermore, the results provide insight into the role parents might play in helping their children cope with their feelings.

## 1. Introduction

Global climate change has been identified since early industrialization [1], although in more recent years, scientists are raising the alarm as the effects of climate change accelerate faster than initially anticipated [2]. The United Nations defines climate change, previously called global warming, as long-term variations in weather patterns and temperatures [3]. This phenomenon includes variations in extreme temperatures, hot or cold, causing acute weather events, rises in sea levels, and extinction of certain animal species [4]. The Intergovernmental Panel on Climate Change (IPCC) has affirmed that anthropogenic (human-made) climate change is undeniable [5]. Messages of urgency and threat now dominate public discourse [6]. Amid the COVID-19 pandemic, children are growing up in an uncertain world and must learn to adapt to the unpredictability of climate change, which can be a burden on their mental health and generate distress [7]. However, very little literature has explored children’s experience of eco-anxiety. Therefore, the main goal of this study was to gain insight into the ways in which children experience eco-anxiety using qualitative methodologies and semi-structured interviews. We also aimed to capture parents’ awareness of their children’s concerns through a questionnaire.

### 1.1 Eco-anxiety

**Fig 1:**
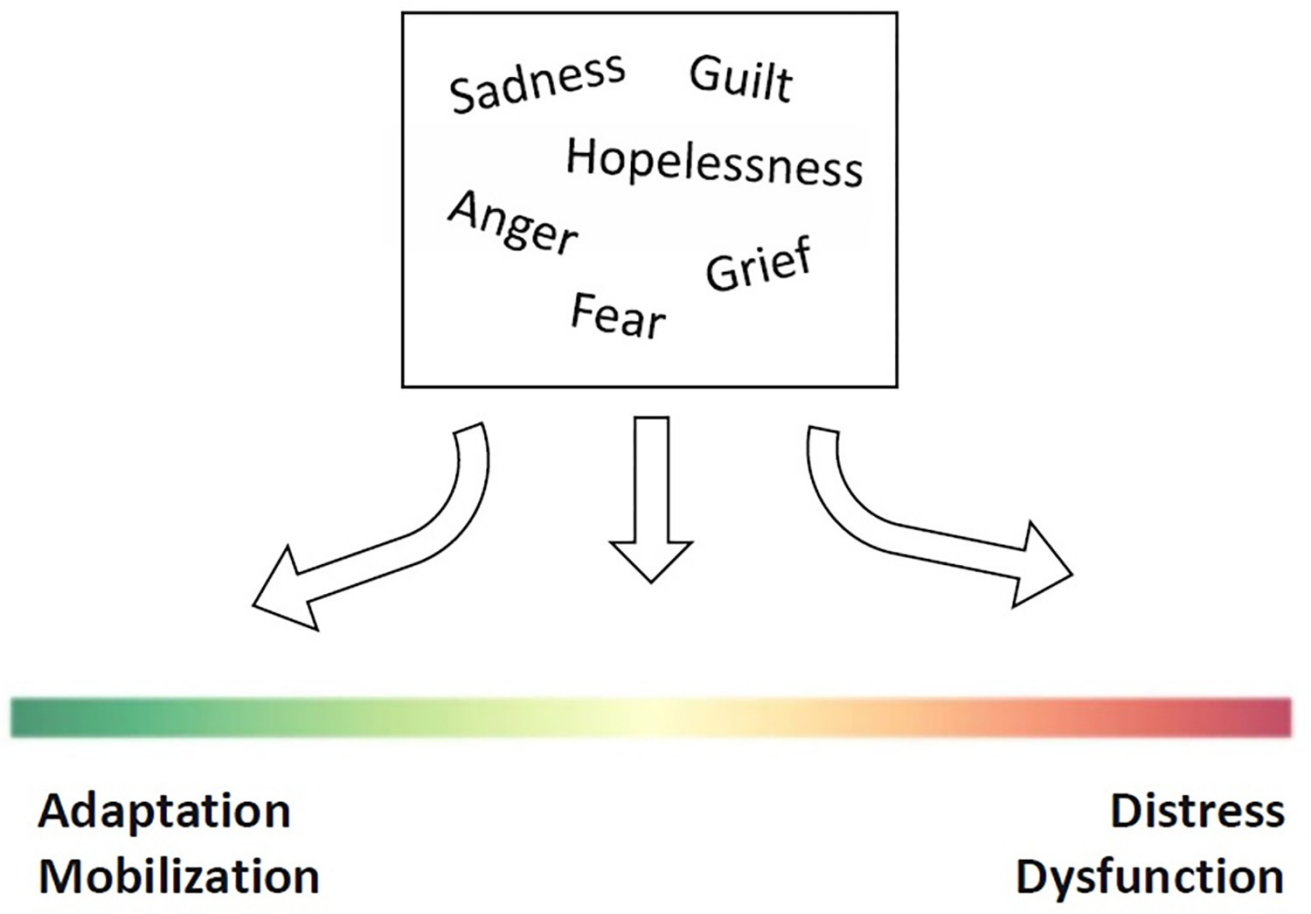
Spectrum hypothesis of eco-anxiety.

Emerging research on eco-anxiety has tended to focus on the mental health effects of being aware of the issue of global climate change, rather than experiencing its direct consequences (such as being the victim of an extreme weather event). The former have been identified as *vicarious* mental health impacts, which are the emotional reactions to observing the effects of climate change happening elsewhere, either through the media or other information sources [8–11]. The term eco-anxiety has been used to define these vicarious emotional reactions. Various authors argue that eco-anxiety’s mental and emotional experiences include an extensive range of feelings [12, 13]. The present paper has operationalized eco-anxiety based on work by Gousse-Lessard and Lebrun-Paré [14], as a psychological state of uneasiness and apprehension in the face of the uncertainty of the future, in the context of climate change. Anxiety may be a central component of this concept, but other emotions such as guilt, sadness, hopelessness, and anger are also included [15, 16]. Nonetheless, an important distinction between clinical anxiety and eco-anxiety must be made, as eco-anxiety is not considered a pathological problem needing a mental health diagnosis. Furthermore, many contend that experiencing these emotions is a normal reaction to the significant threat that is posed by the climate crisis. In several instances, feeling some form of eco-anxiety may be a driving force for action and engagement [12, 16, 17]. However, in some individuals, these feelings may generate debilitating distress, nearing pathological depression or anxiety [18–22]. For this reason, it has been suggested that eco-anxiety could be considered on a spectrum (see Fig 1), ranging from, on one end, emotions leading to adaptive action and mobilization, and on the other end, emotions leading to debilitating paralysis, denial, and dysfunction in daily life [12, 23]. It has been suggested that people may move back and forth along this spectrum [11]. Eco-anxiety is mostly experienced by people with strong ties to nature, environmental scientists, farmers, and youth [24, 25]. However, most studies to date have studied such eco-anxiety in adults. Very little research has examined children’s experiences of eco-anxiety, particularly using rich qualitative methods to provide information on their own perspective of these concerns.

### 1.2 Eco-anxiety in children

Youth are increasingly aware of the issue of climate change, and many are involved in climate movements [26]. For example, the youth activist Greta Thunberg and the “Fridays for Future” movement mobilized many thousands of youths from around the world, giving them a voice and involving them in the fight against climate change [27, 28]. This increased awareness may also be associated with significant worry about the future [29].

Initial evidence of eco-anxiety in children is sparse. However, two recent scoping reviews indicated that youth and children experience emotional reactions in response to the awareness of climate change, which constitutes a form of eco-anxiety [30, 31]. One of the most recent large-scale surveys of young people between the ages of 16 and 25 years across many different countries found that more than half (60%) of respondents were “very” or “extremely” worried about climate change, and 56% believed that “humanity is doomed” [25]. Interviews with youth between 14 and 18 years old revealed that this age group may feel powerless and fear that the climate change situation is out of control, as well as experience strong feelings of anxiety, sadness, guilt, frustration, and anger [32]. Similarly, Strife [33] conducted in-depth interviews with 50 children ages 10–12 years in the United States and found that that they expressed fear and pessimistic feelings about the future of the planet. To our knowledge, it remains one of the only studies that explored the theme of eco-anxiety with younger children. A research gap exists regarding the ways in which different samples of children may be experiencing eco-anxiety, especially using qualitative data to gain an in-depth understanding of their perspective of the issue.

Studies that have examined eco-anxiety in children have explored the ways in which they cope with these emotions. For example, Ojala, has explored how Swedish youth and children cope with worry about climate change through cross-sectional studies [34–36]. Three categories of psychological coping mechanisms have emerged in reaction to climate change. First, problem-focused coping, which involves active concrete action to deal with the stressor, for example searching for information and accomplishing pro-environmental action. This type of coping uses individual action in a more prescribed way and may not be sustainable over time [17]. Second, emotion-focused coping, which entails reducing the negative emotions elicited by climate change, either through de-emphasizing the threat, denial of the situation, distraction, avoidance, or through social support. Finally, meaning-focused coping strategies have been found to be the most adaptive, as they acknowledge the negative emotions and the complexity of the situation while also eliciting “positive” emotions through cognitive reframing (for example, hope and pride). Findings from Ojala’s research revealed that children most often used emotion-focused strategies to cope with the reality of climate change. For example, children’s primary reaction is to avoid thinking about the issue, often by changing the subject or denying the reality [34]. Meaning-focused coping, though less common, is of interest because it has been associated with higher levels of hope and positive affect that can empower autonomous pro-environmental behaviour in children [35]. Work still needs to be done to better understand how young children across different countries and setting use different coping strategies in the context of eco-anxiety.

### 1.3 Parents’ role in children’s eco-anxiety

Children’s awareness of climate change stems from knowledge they obtain from school, the media and from their parents [37]. Research to date on eco-anxiety in children has mostly focused on the role of the school, leaving an important gap in the knowledge on the role that parents may play [38]. The role of parents could be further investigated, as they may contribute information on their children’s experience, as well as insight on the tools needed to promote their children’s mental health in the context of such concerns [39, 40]. However, children are also attuned to their parents’ emotions, and thus, much like anxiety in general, eco-anxiety could be “contagious” [41, 42]. Interestingly, initial evidence suggests that there may be a bidirectional association whereby children’s eco-anxiety can also contribute to parents’ concerns about the planet [43]. Finally, it has been shown that pro-environmental behaviour and values have been associated with climate change worry in adults [44]. Thus, the development of eco-anxiety in children may also occur within the context of the family whereby parents may play a role in the development of these emotions in children, as well as in reducing the child’s distress by working through their own worry and attitudes. This initial evidence suggests that parent’s role may be explored in terms of knowing what their own level of eco-anxiety is, their understanding of their child’s concerns, and/or strategies to help the child cope. However, there are no studies to our knowledge investigating eco-anxiety in parents and their children.

### 1.4 Research contribution and study aims

It is primordial that the children and youth do not develop paralyzing anxiety that could inhibit pro-environmental action and take a toll on their mental health. Hence, it is essential to study children’s emotional reactions to climate change, and the role of parents in shaping these. The climate crisis can only be resolved globally, especially with drastic changes in the lifestyles of those living in “western” countries, including families. With eco-anxiety leading to action paralysis, these changes could be hindered. As such, by studying eco-anxiety, we can help children develop healthy coping mechanisms that will support behavioural changes [45]. The aim of gaining a better understanding of eco-anxiety in children is to first promote their mental health, and second, enable them to act to mitigate the effects of climate change in the long run. Building on findings from prior cross-sectional research, this paper contributes to the research on eco-anxiety in children by providing in-depth descriptions of their emotional experience of climate change awareness, their coping mechanisms, and the role of their parents (own emotions, understanding of children’s concerns, and strategies to help their child cope).

This paper will follow the structure of the consolidated criteria for reporting qualitative research (COREQ) [46]. First, methods are presented to provide information about the study design, recruitment and participants, research procedure, and coding. Next, the results are reported to highlight the major themes found within the interviews with the children and the open-ended questions of the parent questionnaire. Finally, the discussion relates these findings to previous research and interpretations are generated.

## 2. Method

### 2.1 Study design

The present study used a well-established descriptive qualitative methodology to address the paucity of research on how children experience eco-anxiety and the role of their parents [47]. This type of methodology is preferred for emerging topics to gain in-depth understandings on a given issue [48]. Furthermore, since eco-anxiety has a strong subjective nature, a descriptive design was used to probe participants’ experience of the phenomenon. Data was collected separately for children (via interview) and their parents (via questionnaire) to avoid the possibility that the presence of parents could influence interviews with children [49]. The study’s design builds on previous research on eco-anxiety with children [33, 34], while adding a parent component, in order to further investigate how they understand their child’ lived experiences and help them cope with their concerns.

### 2.2 Recruitment and participants

This study was conducted in the province of Quebec, Canada. Quebec has been relatively spared from the direct impacts of climate change, but there are increasing instances of forest fires, floods and extreme temperatures within the province [50]. To be eligible, children had to be between 8 and 12 years old. This age range corresponds to grades 3 to 6 in elementary school, where the curriculum mandates educators to teach the “impacts of human activity on the environment (exploitation, resources, pollution) [51]. Parent-child dyads were recruited using a non-probabilistic convenience sampling technique. Recruitment material was shared via email with different organizations that worked with families in the province of Quebec. Specific organizations working with diverse communities were contacted to approach families from various ethnic identities and socioeconomic statuses, as well as pro-environmental organizations. We did not calculate a refusal rate given that we did not recruit by sending direct messages to potential participants. The sample size was determined *a posteriori* on the basis of information power principles in qualitative research [52]. Hence, when repetition in codes and concepts was reached during thematic analysis, five subsequent confirmatory interviews were conducted, leading to no new codes.

The final sample was composed of 12 families, with 12 parents and 15 children, as some of the families included more than one child in the targeted age range. Most families lived in urban areas (*n* = 8) such as the cities of Montreal and Sherbrooke, three lived in the suburbs (*n* = 3), and one lived in a rural area. Nine of the children identified as girls, and six as boys. Most of the parents identified as women (*n* = 9), two as men and one parent identified as non-binary/genderfluid. Parents were asked about their ethnicity, and 11 identified as White Canadian, one person preferred not to answer. Participant characteristics are presented in Table 1.

**Table 1:** Sample characteristics.

Characteristic	Number
Child age	
8	2
9	1
10	9
11	4
12	2
Child Gender	
Girl	9
Boy	6
Parent gender	
Woman	9
Man	2
Non-binary/genderfluid	1
Parent’s ethnicity	
White Canadian	11
Prefer not to answer	1
Language of interview	
French	13
English	2
Urbanity of family residence	
Urban	8
Suburban	3
Rural	1
	*n*_*Parents*_ = 12
	*n*_Children_ = 15

### 2.3 Procedure

After obtaining parental consent for their own and their children’s participation, parents completed a 10-minute online questionnaire using LimeSurvey on a secure university server. The questionnaire package for parents included sociodemographic information, two validated scales and four open-ended questions. The variables of environmental attitudes and climate change worry were assessed because they were hypothesized to be related to children’s experiences of eco-anxiety. The Climate Change Attitudes Survey developed by Christensen and Knezek [53] was used to assess parents self-reported environmental attitudes. This validated scale is composed of 15 questions on a Likert-type scale ranging from 1 (strongly disagree) to 5 (strongly agree) to obtain a mean attitude score, with higher scores representing higher pro-environmental attitudes. Items include questions such as *“Global climate change will impact future generations”* and *“Human activities cause global climate change”*.

To assess parents self-reported worry, we used the Climate Change Worry Scale developed by Stewart [54]. This validated scale is composed of 10 items on a Likert-type scale ranging from 1 (never) to 5 (always) to obtain a mean worry score for each participant, with higher scores representing higher climate change worry scores. Stewart demonstrated good convergent validity with the Penn State Worry Questionnaire (PWSQ) and The Fear of Weather Scale (FOWS), a high internal reliability (α = 0,95), as well as a high test-retest reliability (r = 0.91, *p* <0.001). Items include *“Thoughts about climate change cause me to have worries about what the future may hold”* and *“Once I begin to worry about climate change*, *I find it difficult to stop*.*”* Reliability of the scale in the present study was not calculated given the small sample size. Parents were also invited to write down the three words that best described how they felt about climate change. In addition, we included open-ended questions to assess parents’ perceptions of their child’s climate change awareness and their role in this.

To explore children’s experiences of eco-anxiety, we used semi-structured interviews—a method that provides space for participants to express themselves in their own words, and to reflect on their own ideas, while also allowing the interviewer a certain flexibility in asking questions [55]. The interviews were conducted in French or in English, based on the children’s preference, between September 2021 and January 2022. Since data collection took place during the COVID-19 pandemic, all interviews were conducted online using an encrypted video-conferencing platform on the first author’s (TLG) university’s secure server. The interviews, conducted by the first author (TLG), lasted between 30 and 40 minutes, were recorded and then transcribed, removing any identifiable information. This interview length was aimed at ensuring the children remained focused and interested. Children were asked to be alone in a separate room from their parents for the interview.

The semi-structured interview guide was created based on an extensive literature review and following the method proposed by Sylvain [56]. In preparation for the interviews and to facilitate the conversation with participants, children were asked to prepare a drawing of their vision of the Earth when they will be adults. Hence, every interview began with the child and the interviewer discussing the drawing as a shared task to establish a relationship [33, 55]. This drawing was not formally analyzed; rather the child’s verbal description of the drawing was used.

Children were asked if they had ever heard about climate change and if they could explain it to someone who had never heard about this issue. Children who said they had never heard about the concept or were not sure how to explain it, were invited to read a short comic strip explaining the issue with the interviewer to gain a better understanding [57]. This comic strip was developed by Élise Gravel, a children’s author and illustrator, and was designed to explain climate change in a child-adapted manner based on scientific facts. It was found on the author’s website, free for non-commercial use. The comic strip provided a gateway to explore the emotions of children who had little to no previous knowledge about climate change. We decided to include those children who were unaware of climate change in the sample, because it provided an opportunity to consider different experiences with climate change.

Each participating child received a $20 gift card to thank them for contributing to this research project. This study was conducted according to the guidelines of the Declaration of Helsinki and approved by the Research Ethics Committee of Bishop’s University. Informed written consent from parents and verbal assent from children was obtained for all subjects involved in the study. Special care was taken to explain to the children the research process and their rights within the research.

### 2.4 Coding and data analysis

All analyses were done in English by fully bilingual researchers. Segments that were chosen as direct quotes were translated by the first author (TLG), maintaining as much as possible the wording and expressions used. Information power was reached after a total of 15 interviews. The principles of information power were indicated by the repetition of certain concepts and no further emerging codes [52]. As such, the depth and range of the concepts being explored seemed to be wholly developed, and the relationships between categories or concepts could also emerge [52].

For the first objective of exploring children’s emotional reaction to the awareness of climate change, reflexive thematic analysis using the method proposed by Braun and Clarke [58, 59] was chosen. It was used to analyze the transcribed interviews and to make sense of the ways in which eco-anxiety was experienced by children, using the qualitative data analysis (QDA) software QDA Miner (v6.0.10). We followed Braun and Clarke’s 6 steps to engage with the dataset: (1) familiarization with the data; (2) generation of initial codes; (3) search for new themes; (4) review of themes; (5) definition and naming of themes; and (6) production of the report [58]. More specifically, in the first phase, two researchers (TLG, AB) read and re-read the transcripts to become immersed and intimately familiar with the content, while taking note of initial observations and ideas. In the second phase, these two researchers independently coded the data to ensure the credibility and trustworthiness of the process. This phase consisted of generating labels or codes to capture important features and address the research question. It required multiple rounds of coding and began after the first interview. The third phase built off these codes to generate initial themes by examining the codes and understanding the broader pattern underlying these codes. When disparity between themes for the two coders existed, they met and discussed the themes and concluded on an agreed-upon set of themes in consultation with the other authors. The fourth phase consisted of developing and reviewing the themes using a master table of themes. From this phase, an initial codebook and a coding tree were generated, and a third reviewer (KH) went over the coding schema and one interview to ensure the codes were thorough.

Throughout these different phases, we used an inductive and deductive approach to data coding, mainly allowing the codes and themes to emerge from the data without any pre-identified concepts. We also used the theory of coping proposed by Ojala [34] to deductively guide a part of the analysis. In the fifth phase, the first author (TLG) did a detailed analysis of each theme, finalizing their label and definition. In the final sixth phase, the first author (TLG) wrote the report of the analysis, contextualizing the findings. Although these phases are presented as sequential, the thematic analysis method was iterative, and it was common to go back and forth through different stages. Throughout the process, the first author (TLG) also used a reflexive journal to explore thoughts, emotions and assumptions that may have arisen during the research and to take note of the process [60].

For the second objective, which was to describe the experience within families, the underlying content was interpreted employing a summative content analysis method, to both count occurrences of certain words and compare themes within families [61]. Analysis started by identifying word frequencies from the questionnaires filled out by the parents in order to highlight the words that were used to represent their emotional experience of climate change. This same process of content analysis was employed to draw out the words used by parents to describe their role in their children’s experiences of climate change. Finally, the experiences of children and parents (within a family) were compared using the content of both children’s interviews and the parents’ questionnaires. Initial open codes identified from the textual data (manifest content) were sorted into categories and then summarized into a table to address the research questions. Again, two researchers (TLG, CMH) went over the data for the reliability of the coding.

The overall results were sent to the families as an infographic for member-checking, in order to offer them an opportunity to discuss the findings and add any information either by email, phone or video conference. None of the families added any information.

## 3. Results

### 3.1 Objective 1: Exploring children’s emotional experiences of climate change

Three overarching themes emerged from our thematic analysis of children’s interviews and are discussed below: 1. children’s knowledge of climate change; 2. their emotional reactions to climate change; and 3. ways of coping with these emotions. Participant quotations can be found in the Tables 2–4.

**Table 2:** Children’s understanding of climate change.

Example quote	Codes	Category
“I would say, there are temperature changes, already, that it increases each year of… well I don’t know how much every year, but I know that it increases every year. There are countries that are less affected than others by climate change, and it’s all because of pollution. From what I hear, there are more greenhouse gasses or something like that…”–Participant 2, 11 years old.	Changes in temperature; Cause is pollution; Greenhouse gasses	General understanding of climate change
“It’s that… there is… it changes, like it rains, it snows, it’s nice, it’s less nice. Climate is just means that, like… is it something else than heat? Like it gets colder when it’s cold and it gets warmer when it’s hot, and sometimes it rains and snows…”–Participant 5, 9 years old.	Confuses with the weather	
“I probably heard about it, but I can’t remember…”–Participant 6, 10 Years old. “Well, I know that it’s been a long time that climate change is there, but um… I don’t know… I don’t talk about it often.”–Participant 1, 8 years old.	Could not define it	
“Well, it’s actually climate changes related to hotter climate, like that affects, well… Plants’ lives and animals’ lives, for example… Glaciers are melting, also coral is dying. There are also species that lose their habitat, well, because either it’s too hot or… there are too many forest fires. So yeah.”–Participant 14, 12 years old.	Effects on biodiversity; Glaciers melting; Loss of habitat; Forest fires	Understanding of the consequences of climate change
“Well, for example, one of my friends said that it sounds like the sun is going to get closer to our Earth and will get bigger…”–Participant 4, 10 years old.	Sun expansion and proximity increase	Misconceptions about climate change
“It’s that people pollute the Earth, and it gets warmer and at some point, it’s going to melt [the Earth].”–Participant 11, 10 years old.	Earth is going to melt	
“I drew little ecological things, like, which sources of energy we were going to use, but it’s for sure that we will not be completely just electricity, from the sun or the wind, but it will also be um… well less fossil energies, but still a little bit, since it takes us a long time to adapt to new energies… We use a lot more energy, but gas and plastic and all the things that pollute are still used and especially for everyday things, like for example a car or a train or just everything like that… I really can’t imagine if there was no fossil energy… Um… I don’t know if everything would have existed…”–Participant 9, 11 years old.	Positive changes	Perception of their future
“Pretty much the same thing with more electric cars…”–Participant 10, 8 years old.	No change	
“There’s a bit of black inside [the Earth]. Like if it was polluted with all the garbage that we throw out just like that. And instead of the green on the Earth, there is just black because little by little, um… Forests are cut to put condos towers.”–Participant 3, 11 years old. “There is so much greenhouse gas that it… that it heated up the Earth so much that is it on fire now. [Okay, so when you’ll be an adult, you think that it may be possible that Earth is really done, and that it burns?] Not done, but that it’s on the verge to…”–Participant 11, 10 years old.	Negative changes	

**Table 3:** Children’s emotions in response to climate change.

Example quote	Coded emotion	Emotion category (Plutchik)	Object of emotion
“Hm… I’m not sure how I feel. (pause) I’m not sure because I hear about it less here. What I hear is that there are forest fires elsewhere, but here I don’t hear about it… that’s why I’m not sure… I’m not sure what I feel…”–Participant 2, 11 years old.	Neutral/uncertain	None	None
“Well, I feel happy and… Well, I also feel grateful that I can… That I’m not in one of those places where it really is a big problem because it’s really hard when that happens, and I’m grateful that… Well, I’m really lucky that I still survive and that it’s kinda still easy for us, for my family.”–Participant 7, 10 years old.	Hope	Apprehension–trust	That people will change. Grateful that they are not living the effects yet.
“[Do you find it a bit anguishing to know that or to think about that—not having kids because it may be the end of the world?] Yeah, I’m like, why now? Why is the world done like that”–Participant 11, 10 years old.	Preoccupied	Apprehension–Fear	Of a natural disaster. That things get worse. That animals go extinct.
“Yeah, sometimes I feel overwhelmed, but more like… it’s like a lot of things to think about… So yeah, I try not to think about it, and then sometimes, I’m like ok well it would be nice to just stop, but it’s like really hard.”–Participant 9, 11 years old.	Overwhelmed	Apprehension–Fear	
“Well, that [people and animals are affected by climate change] makes me feel sad, but because like the humans, they’re not… They are still continuing to do… Like pollute. But not all of them are trying to help the planet.”–Participant 8, 10 years old.	Sadness	Sadness	That the Earth is dying. That nature is dying. That animals are suffering and dying. That people are suffering and dying. For other countries that are suffering because of climate change.
“I’m angry… but I just find it too bad that generations before didn’t think about the future… Just to think about the world now and the world after.”–Participant 2, 11 years old.	Disappointment	Sadness–anger	That people are not acting. That people are not listening. That some people are not aware. That other generations did not think of the consequences of their actions.
“Yeah, I’m also angry at previous generation because actually, it’s us, it’s us who have to live with this… Like people who are 70 will die in like 30 or 20 years even, you know… it’s me who is 11 years old that will live in 40 years, 50 years, 60 years… Probably I’m going to live that again and again because others who are 70 years old today will have passed away when I’ll be 70 years old… But I will be there, I will see everything that degrades.”–Participant 15, 11 years old.	Discouraged	Sadness–anger	
“Anger because I don’t really accept that humans are hurting the planet.”–Participant 4, 10 years old. “Sometimes, I’m just mad and there’s nothing that really can make me feel better, you know.”–Participant 7, 10 years old. “I’m angry because all that, it’s because of pollution! It’s us that do it! And I would really like us to stop that.”–Participant 12, 10 years old.	Anger	Anger	That people are hurting the planet and the animals. Towards people that pollute. Towards past generations. That they will have to live the consequences.
“When I say that [the Earth is going to burn], it makes me scared, but talking about it like this makes me feel better.”–Participant 11, 10 years old. “We [our generation] are scared, but we’re not like terrified either.”–Participant 11, 10 years old. “I’m also kinda scared because… I’m scared that like, the, the, the Earth… Like the Earth goes out… Well, not that it goes out but like… (silence) … yeah.”–Participant 12, 10 years old. “Also, I’m kinda… not scared… But like… I fear a bit that it becomes no longer livable…”–Participant 2, 11 years old.	Fear	Fear	That the world ends. That animals will die. That their life will become difficult because of CC. That even if we stopped polluting now, it would take years to recover.
“I find that a bit… not scary, but… I’m a bit scared of what could happen […] for everyone around… So that, I don’t know everything burns and that it makes even more pollution… That’s what I’m afraid of.”–Participant 2, 11 years old.	Worried	Fear–Apprehension	
“It’s a little bit, just like thinking what’s going to happen, and if it’s nothing if the whole like the… Well, the ice melts, and then there’s no polar bear. No… Most of the animals are dead… So sometimes it is kind of… well… Scary”–Participant 7, 10 years old.	Angst	Fear–Apprehension	
“It really stresses me out a lot… So, yeah, it adds stress. [What stresses you out?] Well, it’s especially the changes in habitat… and that even if we stopped polluting like now on the giant planet… even if we did nothing… actually it’s going to last years.”–Participant 14, 12 years old.	Stress	Fear–Apprehension	

**Table 4:** Children’s coping mechanisms in the context of climate change awareness.

“Well, most of the time, well, either I’m more careful, of course, but actually, especially, to feel better, like sometimes after school, I go walk around, […] and I see junk everywhere, like pieces of plastic […] and I find that disgusting. […] And we just like pick up what we see and put it in the garbage.”–Participant 15	Action	Individual and collective action	Problem-focused coping
“Well, [what gives me hope] I think it’s that more and more people are being careful for the planet because they are becoming aware that it’s really dying now…”–Participant 12	Seeing others act		
“Either I do nothing, and I just check another video, like a little more happy to say okay, it’s all good, I know that now, bye bye the emotion.”–Participant 15	Think of positive things	Avoidance–Distancing	Emotion-focused coping
“I usually don’t think about it [something sad related to climate change] too long. And then… well… I wish I could like stop, to tell the world like to change, but it’s hard, not everyone would like it if I told them stop eating your juice, there’s a lot of plastic that’s used, it kills turtles… it would be more like weird, it would be like… okay, then he would continue… Uh then I change the subject because I don’t think about it too much. And then I know I should, but since I don’t know that…”–Participant 9	Think about something else		
“Well, I felt really sad, but I don’t remember doing something. It was just really, really sad. [Yeah, you just really felt sad. Did you let it out and talk about it or cry a little?] No, I think I kept it in.”–Participant 7	Doing nothing–swallowing up emotions		
“[I would tell a friend] Not to worry, we still have a lot of time to live.”–Participant 11	Telling themselves it’s in a long time	De-emphasize the seriousness of the threat	
“Well, that we are not affected right away, so we should enjoy it… yeah enjoy it.”–Participant 1	Thinking to enjoy life while they still can		
“Um… Well, sometimes I talk about it to my parents. Or my sister.”–Participant 8	Talking about it to a parent/family member	Social support	
“Um, I could talk to pretty much anyone that I know because them, they care, they know what to do… They have a heart for the planet, they want to save the planet too, so I could talk to pretty much anyone. The person I’m closest to, like let’s say I’m next to my mom, I would talk to my mom, but if I’m with my friends, I would talk to my friends.”–Participant 9	Talking about it to a friend		
“I just talk about it to someone close and they say that yes it’s horrible, and I don’t know why it really relieves me to talk about it like that”–Participant 15 “Well, I would tell them [a friend] that… It’s… it’s true that it’s really sad…”–Participant 14	Feeling listened to/understood		
“We’re polluting, yes, but we’re polluting less than before. Things are getting a bit better…”–Participant 13	Acknowledge climate change, but reframe it in a more positive manner	Positive reappraisal	Meaning-focused coping
“Yeah, I, I’m hopeful that in a few decades it will be better… that everything will be… well not… there will have been ravages too, but that now it will start to reconstruct. Climate changes will reduce a bit.”–Participant 2	Focus on the fact that climate change will probably be solved in the future; believing we still have time to change/do something	Positive thinking/existential hope	
“[I would feel better] knowing that people stop polluting and that it stops melting… or that a technology that vacuums smoke and that sends it elsewhere…”–Participant 11 “There are measures being put into place… like boats that go and get garbage…”–Participant 2	Trust in science/technology	Trust in different sources	
“Mm hmm. Well, I don’t really know, but I think it might get worse or better, but then they’ll, well, people will start to act and that it might, well, get really better. I hope so.”–Participant 7	Hope/trust in humanity		

#### 3.1.1 Theme 1: Children have a general understanding of climate change that is concrete

The first significant theme that emerged was that of children’s understanding of climate change. This global theme covers several sub-themes that surfaced from the children’s interviews understanding of the concept of climate change: understanding of the consequences of climate change, misconceptions of climate change, and lack of knowledge on the issue, as well as their perception of the future and the source of their knowledge of climate change. All the subthemes and associated participant quotes can be found in Table 2.

For their general understanding of the concept of climate change, children identified that it involved the heating of the planet due to human pollution. Many named the concept of greenhouse gasses, but very few were able to define it. They were much more articulate about the consequences of climate change than they were about the general definition of the concept. The named consequences included icebergs melting, forest fires, and the impact on animals, especially polar bears. A few children expressed misunderstandings of the situation that did not follow the current scientific consensus, for example, that climate change is happening because the sun is getting bigger and closer to the Earth or that the Earth is melting. It is important to mention that the children who had these misconceptions were both ten years old and one also had additional knowledge of the consequences of climate change that was accurate (we note that they were in the mid age range of the sample, meaning that these misconceptions were not necessarily among the youngest children). Some children had no knowledge about climate change, saying they “didn’t really know about it”, or got confused between the concepts of climate change and the weather. These children remained unsure of what climate change was, even when a different vocabulary was used, like global warming or pollution. Five participants had never heard about climate change or did not know how to explain it. Of these children, one was eight years old, one was nine, and three were ten, representing the younger age range of the sample. Regarding their understanding of the future impacts of climate change, some thought that the Earth would not change, saying it would stay as it is now. A few children mentioned positive changes that may reduce the effects of climate change, like new technologies, but their discourse also indicated that this might not be enough. Most children thought that things would get worse because of pollution, leading to biodiversity loss. This was often indicated by a discourse highlighting a potential increase of the consequences on the environment. One participant even mentioned that he believed the Earth might potentially reach its “endpoint”. Interestingly, most children did not express that they felt directly affected by climate change. They acknowledged small temperature changes but recognized that there are worse impacts elsewhere in the world. Finally, when asked where they had learned about climate change, all the children mentioned school, but some also brought up their parents and media as sources of information. For example, some participants said they heard about the issue on the radio in the car, by doing their own Google searches, or on YouTube. A few also mentioned that they sometimes talked about climate change and shared information on the subject with their friends.

#### 3.1.2 Theme 2: Children experience eco-anxiety when discussing climate change

The next theme that emerged was the emotions surrounding climate change. These were categorized following the classification proposed by Plutchik [62] that breaks down more complex emotions (i.e., not primary emotions) into broader categories (e.g., sadness, anger). We also made sure to preserve the specific issue that generated the emotions, if there was one, as can be found in Table 3.

We found that children mainly mentioned uncomfortable emotions when asked “how does that make you feel” and after explaining some of the consequences of climate change. However, these emotions seemed relatively mild, and none of them suggested that their emotions were uncontrollable or particularly distressing, either verbally or nonverbally (e.g., no children appeared visibly upset, cried, nor shared they were feeling uncomfortable). Mostly, children expressed sadness, especially concerning biodiversity loss, but also for the suffering of other humans around the world. Their discourse suggested a strong empathy for animals. They understood the suffering of animals and the concept of extinction, which made the children feel a form of grief. This feeling was also exacerbated when they observed the inaction of people in general (e.g., a classmate that does not recycle or governmental inertia).

Children also expressed anger and frustration, often associated with disappointment. This anger was frequently directed towards other people who are unaware of environmental problems and who pollute. Participants were frustrated that these people do not realize the scope of the problem. A few children also mentioned feeling frustrated towards previous generations and angry that their generation must deal with problems caused in the past. They realized that they would bear the consequences of climate change, whereas older adults will not. When they mentioned these previous generations, they did not direct it to anyone they knew (e.g., their grandparents or parents); rather, it was people in general.

A few children also mentioned feeling scared about their future and the uncertainty of what could happen. They were also afraid for other people around the world and, again, for animals who might suffer. They mentioned apocalyptic scenarios, like the whole world burning, and expressed a strong sense of fear associated with these narratives. They were afraid that the world they will live in as an adult might be “worse” and “degraded” compared to now.

Some children expressed anxiety, stress, and being overwhelmed, which were all categorized under the emotion of anticipation. For example, one of the participants mentioned that he was considering not having children because of his concern about climate change and end of the world scenarios. Many of the children also mentioned feeling overwhelmed by the enormity of the situation and feeling powerless. They sensed the situation’s complexity, and that part of the solution would be to make significant adjustments to their lifestyle, but that “we can’t just suddenly stop polluting.” Similarly, a sense of urgency coloured the discourse of the children interviewed. Many said that things should be done now or that it may be too late to act, suggesting we are at a critical turning point.

In addition to these uncomfortable emotions, a few children also invoked some more pleasant ones, especially related to feeling grateful that they were not yet adversely affected by climate change. Some also expressed feeling proud and connected to others when they did something environmentally friendly. However, we noticed a complexity of emotions in participants, as the anger they experienced thinking of people who pollute also remained even when expressing their pride or gratitude, suggesting that these emotions can co-exist.

It is also important to mention that a few of the children simply said they did not know how they felt about climate change, especially when it did not affect them, or the situation was less tangible to them. This was mostly seen in the children that had less knowledge about the issue of climate change.

#### 3.1.3 Theme 3: Children’s use various coping mechanisms in response to climate change awareness

The last theme that emerged was the ways in which the children were coping with their emotions regarding their awareness of climate change. Similar to the coping mechanisms that Ojala [35] has previously identified in children, three categories emerged in our study: problem-focused, emotion-focused, and meaning-focused. These can be found in Table 4 along with the participant quotes.

Many coping techniques fell under the category of problem-focused coping, including planning for and taking direct individual action to tackle the issue of climate change. For example, when asked how they would help a friend experiencing eco-anxiety, participants mentioned that they should do pro-environmental actions and gain more knowledge about the issue. A few children also mentioned that witnessing or knowing of collective action, such as community garbage pickups, gave them hope.

The problem-focused coping category also included individual actions participants completed in order to feel like they were contributing to resolving the issue of climate change. These eco-friendly behaviours mostly consisted of concrete, tangible actions the children could do independently. For example, one of the most widely named actions was to pick up garbage. These actions were frequently based on what their parents had taught them or even imposed behaviours in their household, like composting, reducing the use of the car, or even having an electric car. Other actions included decreasing food waste, reducing water consumption, limiting single-use plastics, taking the bus or a bike to go to school, and educating others on the issue. When asked how it felt to do these actions, most of the children mentioned feeling proud and good about themselves.

Most of the children also used emotion-focused coping techniques such as de-emphasizing the seriousness of the problem, distancing themselves from the issue (either through distraction or avoidance), and using social support from their peers and parents. For example, many stated that they would tell a friend that “we still have a lot of time,” or just to “stop thinking about the issue” if they felt “bad.” One participant mentioned that it made her feel better to know she was not alone in experiencing these emotions. Using these emotion-focused techniques generally relieved the children of their unpleasant emotions.

What could be considered meaning-focused coping strategies were evoked relatively infrequently in our sample. One child acknowledged climate change and then reframed it in a more positive manner by highlighting the progress that has been done in the last year to save the environment. Similarly, a few expressed hope in humanity, highlighting that we are polluting less than before, that things are getting better and that, in a few decades, we will have done the right things for the consequences to start being attenuated.

### 3.2 Objective 2: Exploring family dynamics

For our second objective, we used a summative content analysis to document the role of parents in their children’s expressions of eco-anxiety. Each family was viewed as a case to examine commonalities and differences. Parents in this sample all shared high scores on the climate-change attitude scale, indicating that they believed in the issue of climate change and the importance of addressing this crisis (M_Attitude_ = 4.45, SD = 0.36). Worry scores varied, with some parents scoring rather low (around 2.5) and others rather high (above 4) (M_Worry_ = 3.06, SD = 0.55, Min = 2.4, MAX = 4.4). The participants mainly perceived their parenting role as being models for action, educators, and promoters of reflection and hope.

#### 3.2.1 Emotional experiences within families

**Fig 2:**
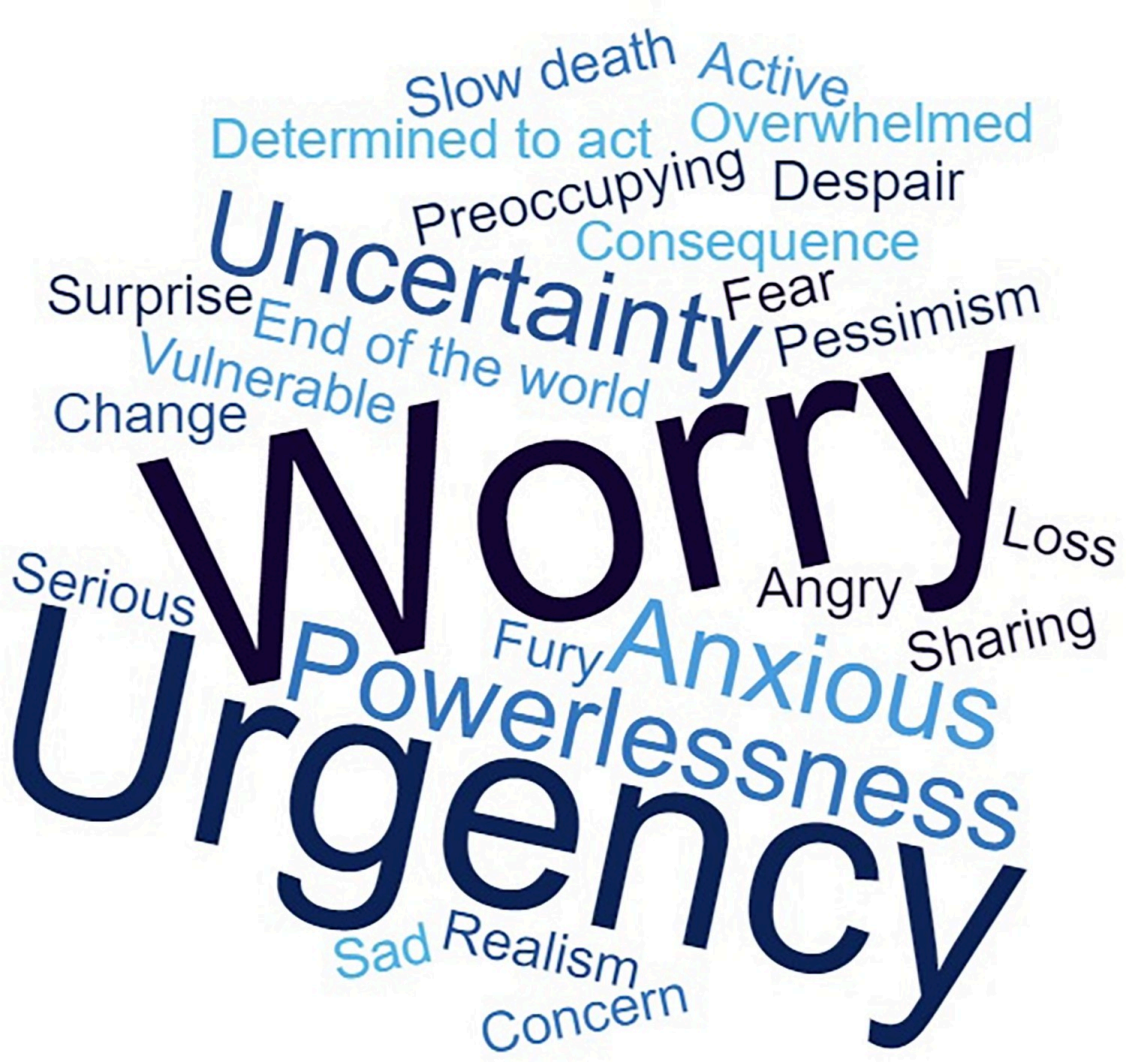
Word cloud of parents’ emotions.

Parents, like their children, expressed a variety of emotions, when asked to list the three that best described the way they felt about climate change. The most common words were worry (7) and urgency (5). These emotions, weighed for their repetition in the questionnaire results, can be found in Fig 2 as a word cloud.

Parents who expressed low levels and higher levels of worry, had children who mentioned emotions such as fear, sadness and anger, as well as being neutral, which did not indicate a clear trend pertaining to parental worry being contagious. A comparison of different concepts for low and high levels of worry in parents can be found in Table 5. The present results do not indicate a clear pattern in the transfer of parental emotions to children, suggesting that eco-anxiety or resilience may develop in children regardless of their parents’ levels of worry. A larger sample would have to be studied to validate the initial findings.

**Table 5:** Comparison of parents with high and low levels of climate change related worry.

Concept	Parents low worry	Parents high worry
Emotions reported by children	Some children mentioned feeling worry, fear, disappointment, frustration, while others felt neutral.	Some children expressed emotions such as sadness, fear, anger, disappointment, and frustration, while others felt neutral.
Belief that their children are preoccupied with climate change	Mixed beliefs about their children’s preoccupation, a few mentioned they did not believe that their children were preoccupied.	All parents who had high worry believed their children were also preoccupied with climate change.
Children’s comprehension of climate change	Mixed comprehension, ranging from low to average.	Mixed comprehension ranging from low to high.
Coping mechanisms reported by children	Mostly individual action (problem-focused), de-emphasizing the threat/denial (emotion-focused), and talking to parents and friends (emotion-focused).	Mixed coping mechanisms including avoidance (emotion-focused), hope in humanity (meaning-focused), talking to parents and friends (emotion-focused), hope in technologies (meaning-focused), and individual action (problem-focused).

Note: the worry scores were obtained with the Climate change 5-point worry scale where mean scores above 3 were considered high, and mean scores below 3 were considered low.

Parents were asked if they believed their child was preoccupied with climate change, and a trend emerged where parents who were themselves worried all believed that their children were also preoccupied, which was the case. Parents with low levels of worry had mixed beliefs about their children’s preoccupations. In this former group, there were a small number of parents who did not believe their child was worried about the issue of climate change, while their child exhibited a form of worry during the interview. In these families, the child tended to mention only one coping mechanism including de-emphasizing the threat/denial, avoidance, or individual action. Overall, most parents, independent of their own worry around climate change, indicated they believed their child was preoccupied with climate change. The interviews revealed that the child indeed had concerns. In these cases, the children often mentioned multiple coping mechanisms, including talking to their parents.

#### 3.2.2 Parents as educators and models

A pattern emerged indicating that the parents perceived their role to be as educators and models around climate change for their children. Indeed, almost all the parents mentioned that they wanted to teach their children pro-environmental behaviours and actions, whether they were themselves worried or not. Some of these parents did have children who demonstrated a greater understanding of climate change. However, when children were asked where they had learned about it, most responded “school,” mainly in their science classes, and only five out of fifteen mentioned that they had heard about it from their parents. Interestingly, some children felt like pro-environmental behaviours were imposed and acted because this was expected from them, not out of eco-anxiety or a larger understanding of their role in climate change. For example, when asked why he picked up garbage and composted, one participant said: “It’s not really me who chooses to do that, so I don’t really know why.”

## 4. Discussion

This qualitative study of children in Quebec, Canada, first explored their awareness of climate change and associated emotional responses (eco-anxiety) through semi-structured interviews. Children who participated expressed emotions that constitute eco-anxiety. As a secondary objective, this study also explored the experiences of the parents of the interviewed children and investigated their role in their child’s eco-anxiety and resilience, as well as potential family dynamics influencing this phenomenon.

### 4.1 Children’s emotional experience of the awareness of climate change

With regard to the emotions evoked by the awareness of climate change, the present study confirms previous findings by Strife [33] concerning children’s expression of sadness, fear, and anger in relation to climate change. Additionally, the manifestation of apprehension, which is a core component of eco-anxiety [14], was present during the interviews. Children expressed feelings of stress related to climate change, and of being overwhelmed by their emotions and fearful for their future, which resembles the experience of youth [25]. Our results also suggest that children’s expression of eco-anxiety resembles that of adults. For example, adults have been found to express fear and worry [63], anger [16], and sadness [64], when asked to describe their eco-anxiety. Furthermore, the focal points of these emotions were similar in both children and adults; they included concern for their own future, the loss of biodiversity, and frustration towards people who do not act [65], although children also demonstrated a unique concern for animals.

An emotion that seems to differ between children and adults was guilt. Indeed, adults tend to feel guilty about their environmental footprint and the state of the Earth they will be leaving for future generations [64, 66]. Although children between the ages of 10 and 12 years old can experience guilt [67], it seems that children in our sample did not express this emotion. This observation is highly relevant in as much as guilt is often used in environmental or governmental campaigns to motivate pro-environmental behaviour. This strategy has shown to be ineffective with adults—guilt without a sense of agency is detrimental to action and can potentially lead to disengagement [66, 68]. As such, greater impact could be had if children receive messages of empowerment through knowledge and action to motivate them to act in helping the planet. Future studies should explore the ways that climate change is addressed in elementary school curriculums. Research with young adults indicated that the lack of integration of affective experiences of climate change may lead students to feel powerless [69].

While emotions felt by the children of this sample were all relatively mild in nature, they were nonetheless experienced as being unpleasant. This differs from other studies that found that some children have significant distress leading them to experience panic attacks or insomnia [70]. Individual differences should be further examined to better understand the factors influencing whether children experience distressing eco-anxiety or, in contrast, the motivation to act. This is true especially given that teachers and parents will address the topic of climate change and risk potentially triggering distressing emotions. Theories in the pedagogy of discomfort are worth considering here as they can be applied in school contexts as well as in families. Pedagogy of discomfort explores emotions that may arise from learning about difficult subjects, like climate change [71]. For example, Boler [72] recommends presenting children with challenging information that destabilizes their world views and balancing this with mechanisms that provide a sense of psychological safety (for example, when teachers acknowledge children’s perspectives and emotions). The children can then step out of their comfort zone without feeling threatened. As such, environmental educators and parents should consider the benefits of creating spaces for the expression of eco-anxiety and other related emotions, thus validating children’s experiences, while providing tools to cope with these emotions, as elaborated by Pihkala [73]. By the same token, as Ojala [71] puts forth, it is also essential that teachers (and parents) explore their own emotions regarding climate change, as their beliefs and perceptions directly impact their teaching approach and how young students respond. Future studies should evaluate the impact of family or school-based programs that address the educative components of climate change while supporting the emotional reactions of children.

As mentioned in our results section, children in this study retain a sense of hope, along with the mild negative emotions discussed earlier. Indeed, previous literature has found that eco-anxiety and worry are associated with feelings of hope in youth [74, 75] and in turn, hope was associated with action [7, 34, 74]. Moreover, another study with young adults found a positive relationship between eco-anxiety and belief in one’s efficacy [76]. Thus, eco-anxiety should not be considered intrinsically problematic, but rather as a natural response. Children should be supported in developing adequate ways of articulating and coping with these emotions, as mentioned above, to avoid significant distress and action paralysis, for example, through mental health promotion programs in schools [73, 77] and in the community [78].

### 4.2 From emotions to action; how to cope with eco-anxiety

Similar to Ojala’s [34, 35] findings, children from our sample mainly used emotion-focused coping strategies in relation to their difficult emotions around climate change, for example, by distancing themselves from or avoiding the problem and using social support; as well as problem-focused coping strategies, predominantly individual action. Although action is essential in moving forward and can be a great source of empowerment [27, 79], caution is warranted on solely relying on individual action to cope because, in the long run, this can lead to burnout and demobilization [29, 80]. Ultimately, coping should rely on emotional awareness [81], finding meaning [34] and social action [82]. Future research should explore the situations in which these different coping methods are used and determine which might be more adaptive in the context of eco-anxiety.

Meaning-focused coping was harder to identify in the present sample. However, a small number of children did suggest acknowledging the complexity of the situation and reframing it in a more positive, attainable way. This reframing shares elements with de-emphasizing the threat and denial but is uniquely characterized by an uncertainty tolerance and nuanced perspective of the future. In other words, it does not mean to minimize the issue. Rather, meaning-focused coping allows individuals to restore a sense of control while acknowledging the stressful situation [83]. This was identified as being most adaptive in the literature, namely because avoiding the subject or acting individually are meant to reduce unpleasant feelings, while meaning-focused coping acknowledges them and evokes pleasant emotions, allowing both to co-exist [35, 84]. Children naturally seemed to be using problem and emotion-focused coping, but since a few hinted towards meaning-focused techniques, this could be an indication that children would be open and sufficiently mature to learn about meaning-focused coping. Although further investigation on potential approaches to support this type of coping is needed.

### 4.3 Parents as models

As mentioned above, parents from this sample all viewed their role as either educators or models for their children in the context of climate change, while some children did not perceive their parents in this way. We noted that parents’ modelling was predominantly done through individual actions, such as composting. Interestingly, very few parents mentioned teaching their children coping skills and sharing their own emotional experiences. This could suggest that the fear and guilt parents experience may lead them to shut down and avoid the issue [85]. As has been suggested by Benoit and colleagues [86], parents should avoid “childism” and openly discuss these difficult subjects with their children.

Interestingly, parents who took part in this study have shown a willingness to begin this discussion but may be hindered by their own emotional reaction to climate change. The literature indicates that parents, along with teachers, are primary socialization agents when it comes to climate change literacy [38]; thus, it is important to better understand and respond to parents’ needs. Future studies should consider developing tools for parents so that they feel equipped to talk about these complex issues with their children and feel at ease dealing with their own emotional responses. Initial evidence indicates that one such tool could be children’s literature, which has the potential to be a powerful resource to introduce the issue to children using age-appropriate stories [87, 88]. Finally, another avenue with interesting potential as a method to help parents, and which has been found to attenuate the complex feelings of eco-anxiety, is spending more time in nature, something that can be done as a family [17, 89]. Connection to nature has a strong positive impact on environmental views in children [90], and forms of ecotherapy in nature have been suggested to promote mental health and give meaning to one’s experience [91]. Hence, future research exploring the benefits of family time in nature on eco-anxiety should be conducted.

### 4.4 Family emotional literacy

Most of the children and parents in this sample experienced some form of worry regarding climate change. Nonetheless, some parents did not think their children were concerned about climate change, when some of them did express worry in the interviews. As such, parents should be aware that their children are potentially preoccupied with the issue, and open the door to discussing these worries, even if they do not believe their child experiences these emotions. This is in line with previous findings that suggest that family discussions of climate change are essential in climate change mitigation in youth [92]. At the same time, it is important to keep in mind that the association between a parent’s and their child’s eco-anxiety should be further investigated using appropriate methodologies to better understand how these are related. Ultimately, this could inform parents that, even if they do not talk about their climate-related apprehension with their children, eco-anxiety could implicitly be passed on to their children, as has been the case for anxiety in general [42, 93].

Moving forward, a perspective of emotional intelligence could be encouraged within families and schools. Emotional intelligence, which, put simply, is one’s ability to recognize and regulate one’s own emotions, has been found to be a mediator in interventions that aimed to reduce anxiety [94], and an overall predictor of positive mental health in children and youth [95, 96]. Parents can be important mentors when it comes to emotional intelligence insofar as their responsiveness to their children’s emotions and their coaching in the identification of emotions are associated with higher emotional intelligence in children [97]. Inversely, in the context of climate change, the perspective of managing or dismissing children’s eco-anxiety can be detrimental to their mental health, as research with youth suggests [25]. As such, promoting introspection and emotional intelligence could support children’s mental health. Adequate support of children’s mental health in the context of the climate crisis, through promoting introspection and emotional intelligence, is only part of the process. Adults should also demonstrate to children that they consider climate change to be a critical issue that needs to be taken seriously [98].

### 4.5 Strengths and limitations

In seeking out families’ perspectives and giving children as well as parents a voice, this study counts notable strengths. Indeed, this research project was important in continuing to explore children’s experiences in the context of climate change, using methods that allowed them to voice their own thoughts and share their lived experiences, as suggested by Strife [33], which was in line with our objectives. Not only do we present the issue from the perspective of the children, but also from the perspective of the parent. Feedback from parents indicated that the study opened the door to a conversation about climate change in their household, for which many were grateful.

In this research project, a method used to ensure the credibility of the qualitative data was having the interviewer (TLG) take the time to build a trusting relationship with the participants prior to beginning the interviews and to express empathy and compassion during the interview to ensure the validity of the discourse [99]. The reliability of the analysis of the qualitative data was safeguarded using independent coders and verification of the conclusions by other members of the research team. The reflective journal, description of the sample, and direct quotations from a variety of participants all ensure confirmability [99].

Despite these strengths, our study also has limitations. Indeed, special attention must be brought to the implications of some of the methodological components of this research project. First, the interviews were conducted online, and the children were in their homes where it was not possible to verify whether the spaces in their houses were entirely confidential. Nonetheless, children all answered positively when asked if they felt like they were in a comfortable and private space before beginning the interview. Although they may have felt more at ease and in control in their own environment, this may have influenced how they answered the questions.

Further, although appropriate for qualitative research, the sampling methods used in this research presented some limitations. While efforts were made, participants were not from diverse ethnic backgrounds. The perspectives of People of Colour may not be represented in this sample, nor those of First Nations communities. Given that First Nations organizations approached for recruitment explained that they only promote research that is solely designed to include Indigenous communities, future research should develop culture-sensitive projects in collaboration with these communities to also explore the emotional experiences of children from these communities that are known to have stronger historic ties with the land and nature (for further reading, see [100–103].

## 5. Conclusion

This study provides a comprehensive view of the emotional experiences pertaining to climate change in children aged 8 to 12 years in Quebec, Canada. It documents preliminary data on the perceived role of parents in relation to their children’s emotional responses to climate change. While children appear to experience emotions such as sadness, anger and apprehension that constitute eco-anxiety, they also reported strategies to manage these difficult emotions, especially when their parents are aware of their concerns. Thus, it is essential to support children’s emotional expression and create spaces to discuss these existential themes. Furthermore, this generation must adapt to a less polluting and carbon intensive world, suggesting the importance of supporting children to move towards the mobilizing side of eco-anxiety and not the paralyzing side. This will include interventions aimed to promote children’s sense of empowerment and agency, as well as meaning-focused coping mechanisms to deal with the normal, yet difficult, emotions of eco-anxiety.

## References

[1] Chao Q, Feng A. Scientific basis of climate change and its response. Global Energy Interconnection. 2018;1: 420–427. doi: 10.14171/j.2096-5117.gei.2018.04.002

[2] Ripple WJ, Wolf C, Newsome TM, Barnard P, Moomaw WR. World Scientists’ Warning of a Climate Emergency. BioScience. 2020;70: 8–12. doi: 10.1093/biosci/biz088

[3] United Nations. What Is Climate Change? | United Nations. [cited 16 Oct 2022]. Available: https://www.un.org/en/climatechange/what-is-climate-change

[4] Hoegh-Guldberg O, Jacob D, Taylor M, Bindi M, Brown S, Camilloni I, et al. Impacts of 1.5°C Global Warming on Natural and Human Systems. In: Global Warming of 1.5°C. An IPCC Special Report on the impacts of global warming of 1.5°C above pre-industrial levels and related global greenhouse gas emission pathways, in the context of strengthening the global response to the threat of climate change, sustainable development, and efforts to eradicate poverty. United Nations Intergovernmental Panel on Climate Change (IPCC); 2019.

[5] Masson-Delmotte V, P. Zhai A. Pirani S., L. Connors C. Péan S. Berger, et al. Climate Change 2021: The Physical Science Basis. Contribution of Working Group I to the Sixth Assessment Report of the Intergovernmental Panel on Climate Change. Cambridge University Press; 2021. Available: https://www.ipcc.ch/report/ar6/wg1/downloads/report/IPCC_AR6_WGI_SPM.pdf

[6] Gills B, Morgan J. Global Climate Emergency: after COP24, climate science, urgency, and the threat to humanity. Globalizations. 2020;17: 885–902. doi: 10.1080/14747731.2019.1669915

[7] Burke SEL, Sanson AV, Van Hoorn J. The Psychological Effects of Climate Change on Children. Curr Psychiatry Rep. 2018;20: 35. doi: 10.1007/s11920-018-0896-9 29637319

[8] Hayes K, Poland B. Addressing Mental Health in a Changing Climate: Incorporating Mental Health Indicators into Climate Change and Health Vulnerability and Adaptation Assessments. IJERPH. 2018;15: 1806. doi: 10.3390/ijerph15091806 30131478 PMC6164893

[9] Berry H, Bowen K, Kjellstrom T. Climate change and mental health: a causal pathways framework. Int J Public Health. 2010;55: 123–132. doi: 10.1007/s00038-009-0112-0 20033251

[10] Cunsolo A, Ellis NR. Ecological grief as a mental health response to climate change-related loss. Nature Clim Change. 2018;8: 275–281. doi: 10.1038/s41558-018-0092-2

[11] Hayes K, Berry P, Ebi KL. Factors Influencing the Mental Health Consequences of Climate Change in Canada. IJERPH. 2019;16: 1583. doi: 10.3390/ijerph16091583 31064134 PMC6539500

[12] Pihkala P. Anxiety and the Ecological Crisis: An Analysis of Eco-Anxiety and Climate Anxiety. Sustainability. 2020;12: 7836. doi: 10.3390/su12197836

[13] Coffey Y, Bhullar N, Durkin J, Islam MS, Usher K. Understanding Eco-anxiety: A Systematic Scoping Review of Current Literature and Identified Knowledge Gaps. The Journal of Climate Change and Health. 2021;3: 100047. doi: 10.1016/j.joclim.2021.100047

[14] Gousse-Lessard A-S, Lebrun-Paré F. Regards croisés sur le phénomène « d’écoanxiété »: perspectives psychologique, sociale et éducationnelle. Éducation relative à l’environnement Regards—Recherches—Réflexions. 2022. doi: 10.4000/ere.8159

[15] Pihkala P. The Cost of Bearing Witness to the Environmental Crisis: Vicarious Traumatization and Dealing with Secondary Traumatic Stress among Environmental Researchers. Social Epistemology. 2020;34: 86–100. doi: 10.1080/02691728.2019.1681560

[16] Stanley SK, Hogg TL, Leviston Z, Walker I. From anger to action: Differential impacts of eco-anxiety, eco-depression, and eco-anger on climate action and wellbeing. The Journal of Climate Change and Health. 2021;1: 100003. doi: 10.1016/j.joclim.2021.100003

[17] Baudon P, Jachens L. A Scoping Review of Interventions for the Treatment of Eco-Anxiety. IJERPH. 2021;18: 9636. doi: 10.3390/ijerph18189636 34574564 PMC8464837

[18] Doherty TJ. Individual impacts and resilience. In: Clayton S, Manning C, editors. Psychology and Climate Change. Academic Press; 2018. pp. 245–266. doi: 10.1016/B978-0-12-813130-5.00010–2

[19] Jones MK, Wootton BM, Vaccaro LD, Menzies RG. The impact of climate change on obsessive compulsive checking concerns. Aust N Z J Psychiatry. 2012;46: 265–270. doi: 10.1177/0004867411433951 22391284

[20] Kaplan EA. Is Climate-Related Pre-Traumatic Stress Syndrome a Real Condition? American Imago. 2020;77: 81–104. doi: 10.1353/aim.2020.0004

[21] Pihkala P. Eco-Anxiety, Tragedy, and Hope: Psychological and Spiritual Dimensions of Climate Change. Zygon. 2018;53: 545–569. doi: 10.1111/zygo.12407

[22] Rosen A. Climate changes are leading to “eco-anxiety,” trauma. International Medical News Group. 2020;48: 10. https://www.mdedge.com/psychiatry/article/221163/anxiety-disorders/climate-changes-are-leading-eco-anxiety-trauma

[23] St-Jean K. Apprivoiser l’écoanxiété et faire de ses écoémotions un moteur de changement. Les Éditions de l’Homme. 2020.

[24] Arcanjo M. Eco-Anxiety: Mental Health Impacts of Environmental Disasters and Climate Change. Climate Institute. 2019 [cited 8 Nov 2020]. Available: http://climate.org/eco-anxiety-mental-health-impacts-of-environmental-disasters-and-climate-change/

[25] Hickman C, Marks E, Pihkala P, Clayton S, Lewandowski RE, Mayall EE, et al. Climate anxiety in children and young people and their beliefs about government responses to climate change: a global survey. The Lancet Planetary Health. 2021;5. doi: 10.1016/S2542-5196(21)00278-3 34895496

[26] Martiskainen M, Axon S, Sovacool BK, Sareen S, Furszyfer Del Rio D, Axon K. Contextualizing climate justice activism: Knowledge, emotions, motivations, and actions among climate strikers in six cities. Global Environmental Change. 2020;65: 102180. doi: 10.1016/j.gloenvcha.2020.102180

[27] Pickard S. “You are stealing our future in front of our very eyes.” The representation of climate change, emotions and the mobilisation of young environmental activists in Britain. E-rea Revue électronique d’études sur le monde anglophone. 2021 [cited 1 Feb 2022]. doi: 10.4000/erea.11774

[28] Sloam J, Pickard S, Henn M. ‘Young People and Environmental Activism: The Transformation of Democratic Politics.’ Journal of Youth Studies. 2022; 1–9. doi: 10.1080/13676261.2022.2056678

[29] Nairn K. Learning from Young People Engaged in Climate Activism: The Potential of Collectivizing Despair and Hope. YOUNG. 2019;27: 435–450. doi: 10.1177/1103308818817603

[30] Martin G, Reilly K, Everitt H, Gilliland JA. Review: The impact of climate change awareness on children’s mental well-being and negative emotions–a scoping review. Child and Adolescent Mental Health. 2022;27: 59–72. doi: 10.1111/camh.12525 34873823

[31] Léger-Goodes T, Malboeuf-Hurtubise C, Mastine T, Généreux M, Paradis P-O, Camden C. Eco-anxiety in Children: A Scoping Review of the Mental Health Impacts of the Awareness of Climate Change, In Press. Front Psychol. 2022. doi: 10.3389/fpsyg.2022.872544 35959069 PMC9359205

[32] Thompson R, Fisher HL, Dewa LH, Hussain T, Kabba Z, Toledano MB. Adolescents’ thoughts and feelings about the local and global environment: a qualitative interview study. Child and Adolescent Mental Health. 2022;27: 4–13. doi: 10.1111/camh.12520 34783152

[33] Strife SJ. Children’s Environmental Concerns: Expressing Ecophobia. The Journal of Environmental Education. 2012;43: 37–54. doi: 10.1080/00958964.2011.602131

[34] Ojala M. Regulating worry, promoting hope: How do children, adolescents, and young adults cope with climate change? IJESE. 2012;7: 537–561.

[35] Ojala M. How do children cope with global climate change? Coping strategies, engagement, and well-being. Journal of Environmental Psychology. 2012;32: 225–233. doi: 10.1016/j.jenvp.2012.02.004

[36] Ojala M. Hope in the Face of Climate Change: Associations With Environmental Engagement and Student Perceptions of Teachers’ Emotion Communication Style and Future Orientation. The Journal of Environmental Education. 2015;46: 133–148. doi: 10.1080/00958964.2015.1021662

[37] Corner A, Roberts O, Chiari S, Völler S, Mayrhuber ES, Mandl S, et al. How do young people engage with climate change? The role of knowledge, values, message framing, and trusted communicators. WIREs Climate Change. 2015;6: 523–534. doi: 10.1002/wcc.353

[38] Pearce H, Hudders L, Van de Sompel D. Young energy savers: Exploring the role of parents, peers, media and schools in saving energy among children in Belgium. Energy Research & Social Science. 2020;63: 101392. doi: 10.1016/j.erss.2019.101392

[39] Baker C, Clayton S, Bragg E. Educating for resilience: parent and teacher perceptions of children’s emotional needs in response to climate change. Environmental Education Research. 2021;27: 687–705. doi: 10.1080/13504622.2020.1828288

[40] Cripps E. Do parents have a special duty to mitigate climate change? Politics, Philosophy & Economics. 2017;16: 308–325. doi: 10.1177/1470594X17709038

[41] Woodruff-Borden J, Morrow C, Bourland S, Cambron S. The Behavior of Anxious Parents: Examining Mechanisms of Transmission of Anxiety From Parent to Child. Journal of Clinical Child & Adolescent Psychology. 2002;31: 364–374. doi: 10.1207/S15374424JCCP3103_08 12149974

[42] Crandon TJ, Scott JG, Charlson FJ, Thomas HJ. A social–ecological perspective on climate anxiety in children and adolescents. Nat Clim Chang. 2022;12: 123–131. doi: 10.1038/s41558-021-01251-y

[43] Lawson DF, Stevenson KT, Nils PM, Carrier SJ, Renee LS, Erin S. Children can foster climate change concern among their parents. Nature Climate Change. 2019;9: 458–462. doi: 10.1038/s41558-019-0463-3

[44] Verplanken B, Marks E, Dobromir AI. On the nature of eco-anxiety: How constructive or unconstructive is habitual worry about global warming? Journal of Environmental Psychology. 2020;72: 101528. doi: 10.1016/j.jenvp.2020.101528

[45] van Zomeren M, Pauls IL, Cohen-Chen S. Is hope good for motivating collective action in the context of climate change? Differentiating hope’s emotion- and problem-focused coping functions. Global Environmental Change. 2019;58: 101915. doi: 10.1016/j.gloenvcha.2019.04.003

[46] Tong A, Sainsbury P, Craig J. Consolidated criteria for reporting qualitative research (COREQ): a 32-item checklist for interviews and focus groups. International Journal for Quality in Health Care. 2007;19: 349–357. doi: 10.1093/intqhc/mzm042 17872937

[47] Doyle L, McCabe C, Keogh B, Brady A, McCann M. An overview of the qualitative descriptive design within nursing research. Journal of Research in Nursing. 2020;25: 443–455. doi: 10.1177/1744987119880234 34394658 PMC7932381

[48] Sandelowski M. What’s in a name? Qualitative description revisited. Research in Nursing & Health. 2010;33: 77–84. doi: 10.1002/nur.20362 20014004

[49] Gardner H, Randall D. The effects of the presence or absence of parents on interviews with children. Nurse Researcher. 2012;19: 6–10. 10.7748/nr2012.01.19.2.6.c8902 22338802

[50] Leclerc L, Siron R, Logan T, Côté H. Sommaire de la synthèse des connaissances sur les changements climatiques au Québec. Ouranos; 2015. Available: https://ouranos.ca/wp-content/uploads/SynthesePartie2.pdf

[51] Ministère de l’Éducation et de l’Enseignement supérieur du Québec. L’éducation au développement durable—Volume 3—L’intégration du développement durable dans l’enseignement. 2019. Available: http://www.education.gouv.qc.ca/fileadmin/site_web/documents/PSG/politiques_orientations/EDD-volume3-Integration.pdf

[52] Malterud K, Siersma VD, Guassora AD. Sample Size in Qualitative Interview Studies: Guided by Information Power. Qual Health Res. 2016;26: 1753–1760. doi: 10.1177/1049732315617444 26613970

[53] Christensen R, Knezek G. The Climate Change Attitude Survey: Measuring Middle School Student Beliefs and Intentions to Enact Positive Environmental Change. International J Sci Env Ed. 2015;10: 773–788. doi: 10.12973/ijese.2015.276a

[54] Stewart AE. Psychometric Properties of the Climate Change Worry Scale. Int J Environ Res Public Health. 2021;18. doi: 10.3390/ijerph18020494 33435348 PMC7826965

[55] Prior J. Practical Research with Children. Taylor&Francis. Van Herwegen J, editor. Routledge; 2016. Available: https://bookshelf.vitalsource.com/books/9781317384045

[56] Sylvain L. Le Guide d’entrevue son élaboration, son évolution et les conditions de réalisation d’une entrevue. Actes du 12e Colloque de l’ARC. 2002 [cited 5 Jul 2022]. Available: https://eduq.info/xmlui/handle/11515/29768

[57] Gravel É. Le changement climatique, c’est quoi? In: Élise Gravel [Internet]. [cited 7 Jul 2022]. Available: http://elisegravel.com/blog/changement-climatique-cest-quoi/

[58] Braun V, Clarke V. Using thematic analysis in psychology. Qualitative Research in Psychology. 2006;3: 77–101. doi: 10.1191/1478088706qp063oa

[59] Braun V, Clarke V. Thematic Analysis. APA handbook of research methods in psychology, Vol 2: Research designs: Quantitative, qualitative, neuropsychological, and biological. Washington, DC, US: American Psychological Association; 2012. pp. 57–71. doi: 10.1037/13620-000

[60] Ortlipp M. Keeping and Using Reflective Journals in the Qualitative Research Process. The Qualitative Report. 2008;13. doi: 10.46743/2160-3715/2008.1579

[61] Hsieh H-F, Shannon SE. Three Approaches to Qualitative Content Analysis. Qual Health Res. 2005;15: 1277–1288. doi: 10.1177/1049732305276687 16204405

[62] Plutchik R. The Nature of Emotions. American Scientist. 2001;89: 344–350. ISSN 0003-0996

[63] Kemkes RJ, Akerman S. Contending with the nature of climate change: Phenomenological interpretations from northern Wisconsin. Emotion, Space and Society. 2019;33: 100614. doi: 10.1016/j.emospa.2019.100614

[64] Ágoston C, Csaba B, Nagy B, Kőváry Z, Dúll A, Rácz J, et al. Identifying Types of Eco-Anxiety, Eco-Guilt, Eco-Grief, and Eco-Coping in a Climate-Sensitive Population: A Qualitative Study. International Journal of Environmental Research and Public Health. 2022;19: 2461. doi: 10.3390/ijerph19042461 35206648 PMC8875433

[65] Soutar C, Wand APF. Understanding the Spectrum of Anxiety Responses to Climate Change: A Systematic Review of the Qualitative Literature. International Journal of Environmental Research and Public Health. 2022;19: 990. doi: 10.3390/ijerph19020990 35055813 PMC8776219

[66] Moore MM, Yang JZ. Using Eco-Guilt to Motivate Environmental Behavior Change. Environmental Communication. 2020;14: 522–536. doi: 10.1080/17524032.2019.1692889

[67] Olthof T, Schouten A, Kuiper H, Stegge H, Jennekens-Schinkel A. Shame and guilt in children: Differential situational antecedents and experiential correlates. British Journal of Developmental Psychology. 2000;18: 51–64. doi: 10.1348/026151000165562

[68] Antonetti P, Maklan S. Feelings that Make a Difference: How Guilt and Pride Convince Consumers of the Effectiveness of Sustainable Consumption Choices. J Bus Ethics. 2014;124: 117–134. doi: 10.1007/s10551-013-1841-9

[69] Jones CA, Davison A. Disempowering emotions: The role of educational experiences in social responses to climate change. Geoforum. 2021;118: 190–200. doi: 10.1016/j.geoforum.2020.11.006

[70] Hickman C. We need to (find a way to) talk about … Eco-anxiety. Journal of Social Work Practice. 2020;34: 411–424. doi: 10.1080/02650533.2020.1844166

[71] Ojala M. Safe spaces or a pedagogy of discomfort? Senior high-school teachers’ meta-emotion philosophies and climate change education. The Journal of Environmental Education. 2021;52: 40–52. doi: 10.1080/00958964.2020.1845589

[72] Boler M. Feeling Power: Emotions and Education. London, UK: Taylor & Francis Group; 1999. Available: http://ebookcentral.proquest.com/lib/usherbrookemgh-ebooks/detail.action?docID=214511

[73] Pihkala P. Eco-Anxiety and Environmental Education. Sustainability. 2020;12: 10149. doi: 10.3390/su122310149

[74] Stevenson K, Peterson N. Motivating Action through Fostering Climate Change Hope and Concern and Avoiding Despair among Adolescents. Sustainability. 2016;8: 6. doi: 10.3390/su8010006

[75] Li CJ, Monroe MC. Exploring the essential psychological factors in fostering hope concerning climate change. Environmental Education Research. 2019;25: 936–954. doi: 10.1080/13504622.2017.1367916

[76] Maran DA, Begotti T. Media Exposure to Climate Change, Anxiety, and Efficacy Beliefs in a Sample of Italian University Students. IJERPH. 2021;18: 9358. doi: 10.3390/ijerph18179358 34501946 PMC8431103

[77] Nice ML, Forziat-Pytel K, Benoit C, Sturm DC. School Counselor and Environmental Educator Partnerships: Reducing Eco-Anxiety From Climate Change, Increasing Self-Efficacy, and Enhancing Youth Advocacy. Professional School Counseling. 2022;26: 2156759X221090525. doi: 10.1177/2156759X221090525

[78] Cunsolo A, Harper SL, Minor K, Hayes K, Williams KG, Howard C. Ecological grief and anxiety: the start of a healthy response to climate change? The Lancet Planetary Health. 2020;4: 261–263. doi: 10.1016/S2542-5196(20)30144-3 32681892

[79] Coppola IG, Gould RK, Seidl A, Fothergill A. Eco-Anxiety in “the Climate Generation”: Is Action an Antidote? Thesis, University of Vermont. 2021. Available: https://scholarworks.uvm.edu/envstheses/71

[80] von Hellermann P. From Ecophany to Burnout? An Anthropologist’s Reflections on Two Years of Participating in Council-Citizen Climate Governance in Eastbourne. World. 2021;2: 521–537. doi: 10.3390/world2040032

[81] Graham P, Kuyvenhoven C, Upitis R, Arshad-Ayaz A, Scheinman E, Khan C, et al. The Emotional Experience of Sustainability Courses: Learned Eco-Anxiety, Potential Ontological Adjustment. Journal of Education for Sustainable Development. 2020;14: 190–204. doi: 10.1177/0973408220981163

[82] Trott CD. Children’s constructive climate change engagement: Empowering awareness, agency, and action. Environmental Education Research. 2020;26: 532–554. doi: 10.1080/13504622.2019.1675594

[83] Park CL, Folkman S. Meaning in the Context of Stress and Coping. Review of General Psychology. 1997;1: 115–144. doi: 10.1037/1089-2680.1.2.115

[84] Folkman S. The case for positive emotions in the stress process. Anxiety Stress Coping. 2008;21: 3–14. doi: 10.1080/10615800701740457 18027121

[85] Ekholm S, Olofsson A. Parenthood and Worrying About Climate Change: The Limitations of Previous Approaches. Risk Analysis. 2017;37: 305–314. doi: 10.1111/risa.12626 27164412

[86] Benoit L, Thomas I, Martin A. Review: Ecological awareness, anxiety, and actions among youth and their parents–a qualitative study of newspaper narratives. Child and Adolescent Mental Health. 2022;27: 47–58. doi: 10.1111/camh.12514 34687125

[87] Léger-Goodes T, Malboeuf-Hurtubise C, Camden C. Aborder l’éco-anxiété avec les enfants dans le contexte de la crise climatique. Communication Jeunesse; 2021 février; Québec.

[88] Benevento SV. Communicating Climate Change Risk to Children: A Thematic Analysis of Children’s Literature. Early Childhood Educ J. 2022 [cited 17 Jun 2022]. doi: 10.1007/s10643-021-01294-y

[89] Asah ST, Bengston DN, Westphal LM, Gowan CH. Mechanisms of Children’s Exposure to Nature: Predicting Adulthood Environmental Citizenship and Commitment to Nature-Based Activities. Environment and Behavior. 2018;50: 807–836. doi: 10.1177/0013916517718021

[90] Hahn ER. The developmental roots of environmental stewardship: Childhood and the climate change crisis. Current Opinion in Psychology. 2021;42: 19–24. doi: 10.1016/j.copsyc.2021.01.006 33636523

[91] Jordan M, Hinds J. Ecotherapy: Theory, Research and Practice. UK: Red Globe Press; 2016.

[92] Lawson DF, Stevenson KT, Peterson MN, Carrier SJ, Seekamp E, Strnad R. Evaluating climate change behaviors and concern in the family context. Environmental Education Research. 2019;25: 678–690. doi: 10.1080/13504622.2018.1564248

[93] Schrock M, Woodruff-Borden J. Parent-Child Interactions in Anxious Families. Child & Family Behavior Therapy. 2010;32: 291–310. doi: 10.1080/07317107.2010.515523

[94] Kwok SYCL, Gu M, Tam NWY. A Multiple Component Positive Psychology Intervention to Reduce Anxiety and Increase Happiness in Adolescents: The Mediating Roles of Gratitude and Emotional Intelligence. J Happiness Stud. 2022 [cited 27 Apr 2022]. doi: 10.1007/s10902-021-00487-x

[95] Ciarrochi J, Deane FP, Anderson S. Emotional intelligence moderates the relationship between stress and mental health. Personality and Individual Differences. 2002;32: 197–209. doi: 10.1016/S0191-8869(01)00012-5

[96] Punia S, Sangwan S. Emotional Intelligence and Social Adaptation of School Children. Journal of Psychology. 2011;2: 83–87. doi: 10.1080/09764224.2011.11885466

[97] Alegre A. Parenting Styles and Children’s Emotional Intelligence: What do We Know? The Family Journal. 2011;19: 56–62. doi: 10.1177/1066480710387486

[98] Sanson AV, Burke SEL, Van Hoorn J. Climate Change: Implications for Parents and Parenting. Parenting. 2018;18: 200–217. doi: 10.1080/15295192.2018.1465307

[99] Bradshaw C, Atkinson S, Doody O. Employing a Qualitative Description Approach in Health Care Research. Global Qualitative Nursing Research. 2017;4: 2333393617742282. doi: 10.1177/2333393617742282 29204457 PMC5703087

[100] Cunsolo A, Harper SL, Ford JD, Edge VL, Landman K, Houle K, et al. Climate change and mental health: an exploratory case study from Rigolet, Nunatsiavut, Canada. Climatic Change. 2013;121: 255–270. doi: 10.1007/s10584-013-0875-4

[101] Hunter E. Radical Hope and Rain: Climate Change and the Mental Health of Indigenous Residents of Northern Australia. Australas Psychiatry. 2009;17: 445–452. doi: 10.1080/10398560903062927 20001364

[102] MacKay M, Parlee B, Karsgaard C. Youth Engagement in Climate Change Action: Case Study on Indigenous Youth at COP24. Sustainability. 2020;12: 6299. doi: 10.3390/su12166299

[103] Middleton J, Cunsolo A, Jones-Bitton A, Wright CJ, Harper SL. Indigenous mental health in a changing climate: a systematic scoping review of the global literature. Environ Res Lett. 2020;15: 053001. doi: 10.1088/1748-9326/ab68a9

